# Influence of first- and second-generation antipsychotics on anthropometric parameters of male psychiatric patients

**DOI:** 10.4102/sajpsychiatry.v28i0.1772

**Published:** 2022-02-17

**Authors:** Maseqhala P. Nkondo-Ndaba, Pierre M. Joubert, Theona Ballyram, Charl Janse van Rensburg

**Affiliations:** 1 Department of Psychiatry, Faculty of Health Sciences, School of Medicine, University of Pretoria, Pretoria, South Africa; 2 Weskoppies Psychiatric Hospital, Pretoria, South Africa; 3 Biostatistics Unit, Medical Research Council, Pretoria, South Africa

**Keywords:** psychiatric patients, antipsychotics, BMI, waist circumference, waist-to-hip ratio, waist-to-height ratio, body measures

## Abstract

**Background:**

The use of antipsychotic medication, particularly second generation antipsychotics (SGAs) is a major risk factor for cardiovascular disease in people with severe mental illness (SMI). Few studies have compared body measures of people with SMI taking first generation antipsychotics (FGAs) to those taking SGAs.

**Aim:**

We compare body measures between long-term male inpatients using either FGAs or SGAs.

**Setting:**

The study was conducted at Weskoppies Psychiatric Hospital, in Pretoria, Gauteng.

**Methods:**

A total of 30 patients were selected from a list of male inpatients and were included in our study. Each participant had the following anthropometric measures done and these were compared between the two groups: Waist circumference (WC), body mass index (BMI), waist to hip ratio (WHR), waist to height ratio (WHtR) and hip circumference (HC). Hospital records were used to record demographic variables, diagnosis, comorbid disease and psychotropic medication for each participant.

**Results:**

Participants in the FGA and SGA groups had similar body measures, resulting in similar BMI, WHR and WHtR. Participants had a mean HC of 100.5 cm, 95% confidence interval (CI) (97.68, 103.22). BMI ranged from 21.87 kg/m² to 37.65 kg/m², with an overall mean of 28.5 kg/m², 95% CI (26.69, 30.22). Participants had a mean WHtR of 0.59, 95% CI (0.56, 0.61). Participants had a mean WC of 100.6 cm and 95% CI (96.26, 104.87), and the mean WHR of both groups was 1.0.

**Conclusion:**

Participants using FGAs and SGAs had similar body measures, and these indicated that this sample of male inpatients with SMI is at high risk for CVD.

## Introduction

People with severe mental illness (SMI), especially those with psychotic disorders such as schizophrenia, reportedly have a shorter life expectancy compared to the general population. This shorter life expectancy is associated with increased rates of risk factors like hypertension, diabetes, obesity, strokes, myocardial infarction, coronary heart disease, peripheral vascular disease, congestive heart failure, as well as other cardiovascular diseases (CVDs).^1,2,3^ Evidence suggests that the incidence of CVD increases with antipsychotic use, higher body mass index (BMI) and higher baseline CVD prevalence.^1^ Second-generation antipsychotic (SGA) use is particularly associated with metabolic syndrome, which is associated with increased CVD risk factors including hypertension, insulin resistance, hypercholesterolemia and obesity.^4,5^

The first antipsychotic, chlorpromazine, was introduced in 1952.^6^ The introduction of first-generation antipsychotics (FGAs) led to significant improvements in the symptoms of schizophrenia, including hallucinations and delusions, but were unfortunately associated with an increased risk for extra-pyramidal side effects.^7^ Aside from schizophrenia, FGAs are also used to treat symptoms of bipolar disorder, such as mania.^8^ Some commonly used FGAs include haloperidol, flupentixol and zuclopenthixol.^9^

Second-generation or ‘atypical’ antipsychotic drugs (SGAs) are compounds that are efficacious against the symptoms of schizophrenia, but with nil or minimal extra-pyramidal side effects.^10^ Clozapine was the first SGA to be described. Its clinical effects were first reported in 1966, and it was first approved in the United States in 1990.^10^ Second-generation antipsychotics are used to treat schizophrenia, bipolar disorder, schizoaffective disorder, major depressive disorder as well as acute agitation or irritability that may be associated with illnesses such as schizophrenia, bipolar disorder and autistic disorder. They are also used off-label to treat anxiety disorders, delirium, dementia and insomnia.^11^ Although SGAs gained popularity because of their reduced risk of extra-pyramidal side effects, evidence from the Clinical Antipsychotic Trials of Intervention Effectiveness (CATIE) study indicates that SGAs may lead to an increased risk of metabolic side effects, including type 2 diabetes, central obesity and hyperlipidaemia in patients with SMI.^12^

Different antipsychotics seem to affect weight gain differently. The SGAs clozapine and olanzapine are associated with the greatest weight gain.^3,13^ Chlorpromazine, an FGA, is also associated with weight gain, while quetiapine and risperidone, both SGAs, have an intermediate risk. Asenapine, amisulpride, fluphenazine, haloperidol and ziprasidone have little effect on weight, while aripiprazole promotes weight loss.^2^ Recommendations suggest that patients should be screened for CVD risk factors when initiating antipsychotics.^14^

In psychiatric settings, a simple measure for screening for CVD risk may be useful, because people with SMI are twice as likely to have metabolic syndrome compared to the general population.^4,5^ Currently, the recommended body measures for the assessment of cardiovascular risk in patients with SMI on antipsychotics are BMI and waist circumference (WC), which are measures of overall adiposity and central adiposity, respectively.^14^ There is still no clarity about which body measures would be the best predictors of CVD risk despite years of research.^15,16,17,18^

Other body measures include anthropometric indices such as waist-to-hip ratio (WHR) and WC, which are measures of central obesity. These measures are both convenient and cost-effective ways of assessing CVD risk and are suitable for use in busy, poorly resourced settings.^15^ Several studies have investigated the value of different anthropometric indices in predicting CVD risk as summarised in Table 1. Increased BMI, WC and WHR were all significantly correlated to 5- or 10-year CVD risk in the Australian Diabetes, Obesity and Lifestyle (AusDiab) Study.^16^ The strongest correlations were observed between WHR and absolute risk estimates.^16^ In Caucasian men and women, WC is a stronger predictor of CVD risk than BMI.^17^ In Chinese people, waist-to-height ratio (WHtR) was a better indicator of dyslipidaemia, hyperglycaemia and CVD risk than BMI, WC, hip circumference (HC), WHR and body shape index (ABSI).^18^ In sub-Saharan men and women, WHtRs of greater than 0.5 predicted risk of CVD.^15^ In adults from Southeast Asia, BMI and WHtR had the best clinical utility in predicting CVD risk in patients overall, even better than a combination of BMI and WC.^19^ In Egypt, CVD risk was best predicted by increased WC regardless of the presence of other comorbidities and general obesity, with men at higher risk if their WC was greater than 100.5 cm and women at higher risk if their WC was greater than 96.25 cm.^20^ As indicated by the studies referenced above, CVD risk is associated with BMI, WC, WHtR and WHR, accordingly, these body measures were used in our study.

**TABLE 1:** Summary of body measures that have been investigated in different populations as predictors of cardiovascular disease risk.

Body measures investigated	Body measures found the highest CVD risk correlation	Population/demographics
WC/WHtR	WHtR	Black Adult South Africans^15^
BMI/WC/WHR	WHR	Adults in Australia^16^
BMI/WC/WHR/WHtR/BFP	WC	Caucasian males and females without CVD^17^
BMI/WC/HC/WHR/WHtR/ABSI	WHtR	Chinese adults^18^
WC/WHR/WHtR/BMI/BAI	BMI, WHtR	Adults in Singapore^19^
WC/HC/BMI/WHR/WHtR	WC	Egyptian adults^20^

CVD, cardiovascular disease; WC, waist circumference; WHtR, waist-to-height ratio; BMI, body mass index; WHR, waist-to-hip ratio; HC, hip circumference; ABSI, body shape index; BFP, body fat percentage; BAI, body adiposity index.

Most of the studies that have investigated the metabolic effects of FGAs and SGAs measured biological markers of metabolic risk, but very few studies have investigated the influence of antipsychotic medication on body measures associated with CVD risk.^13,21,22,23^ Even fewer studies have investigated CVD risk based on body measures in people with SMI, who are on FGAs or SGAs. In our study, we compared body measures of long-term male inpatients on FGAs and those on SGAs to obtain an impression of their risk for CVD and to record possible differences between the two groups. Firstly, we gathered the body measures including WHR, WHtR, BMI, WC and HR of long-term inpatients at Weskoppies Psychiatric hospital, who were on either SGAs or FGAs. Secondly, we assessed if body measures of patients on FGAs and SGAs differed significantly.

## Methods

### Study design

Our study was a cross-sectional quantitative study.

### Setting and sampling

Our study was conducted at a specialised psychiatric hospital in Pretoria. The study population included inpatients with SMI in the chronic care unit (CCU), who had been prescribed either FGAs or SGAs. The authors obtained informed consent from potential study participants.

Participants were selected from a group of male inpatients identified by the treating team of the CCU. Male inpatients in the CCU who were older than 18 years, had been admitted for at least two years and had been prescribed either SGAs or FGAs were included in the study. Potential participants were excluded if they were on a combination of FGAs and SGAs or on other medications associated with weight gain. such as sodium valproate and tricyclic antidepressants. The patterns of body fat depositions in men and women differ^24^ and differences in sex would influence the results. In order to standardise the measurements and interpretation of results, only males were included in our study.

Patients who lacked capacity to consent to take part in our study as well as those who declined participation were also excluded.

### Measurements

Participants were measured in a standing position, without shoes and wearing light clothing. All participants were weighed on the same digital scale. The height was measured using a stadiometer with participants standing against the wall with their feet together. The BMI of each participant was calculated using the formula: BMI = weight in kilograms (kg) divided by height in meters squared (m^2^). All measurements were carried out by the primary investigator. The WC and HC were measured in centimetres using the same stretch-resistant measuring tape. Waist circumference was measured at the approximate midpoint between the lower margin of the last palpable rib and the top of the iliac crest, with the tape snug against the body, but not pulled so tight that it is constricting. Hip circumference was measured as the maximum circumference over the buttocks. Waist-to-hip ratio was calculated as the ratio of WC over the HC. Waist-to-height ratio was calculated as the ratio of the WC over the height. All measurements were performed according to the WHO standards.^24^

We conducted our study during the COVID-19 pandemic and followed all standard COVID-19 protocols:

- Sanitised all equipment between each participant.
- All participants and the researcher wore masks at all times.
- Both authors and participants sanitised their hands before and after measuring.
- Contact with participants was kept brief enough to conduct all necessary measurements.
- The authors and participants kept a distance of at least 1.5 m while explaining the research and obtaining informed consent.

Participant demographics, diagnosis, comorbid disease and their psychotropic medication were recorded from hospital records.

### Data analysis

We described continuous variables using the descriptive mean, median, standard deviation and inter-quartile range. Categorical variables were described using frequencies and proportions, and differences in categorical variables were assessed using chi-square tests. The *t*-test was used to compare mean body measures between the FGA and SGA groups. Tests were evaluated at 5% level of significance. All analysis was done using STATA 15.

### Ethical considerations

Our study was approved by the Faculty of Health Sciences Research Ethics Committee at the University of Pretoria (262/2020).

## Results

The age of the participants ranged from 24 to 74 years, with a mean age of 48.4 years. Participants on FGAs were slightly older (mean = 50.7 years) than those on SGAs (mean = 46.2 years). Age was not correlated to any of the continuous variables. Thus, there was no need to control for age.

All participants in the FGA group (*n* = 15) had been diagnosed with a psychotic disorder, including schizophrenia (*n* = 13), a psychotic disorder as a result of another medical condition (*n* = 1) and a cannabis induced psychotic disorder (*n* = 1). In the SGA group (*n* = 15), participants were diagnosed with schizophrenia (*n* = 13), mild intellectual disability (*n* = 1) and major neurocognitive disorder (*n* = 1).

Participants had the following comorbid conditions, hypertension (FGA [*n* = 3]; SGA [*n* = 3]), diabetes (FGA [*n* = 2]; SGA [*n* = 3]), hypercholesterolemia (FGA [*n* = 3]; SGA [*n* = 3]), HIV (FGA [*n* = 1); SGA (*n* = 1]), benign prostate hyperplasia (FGA [*n* = 1]; SGA [*n* = 0]), cannabis use disorder (FGA [*n* = 2]; SGA [*n* = 1]), chronic obstructive pulmonary disease (FGA [*n* = 1]; SGA [*n* = 0]) and somatisation disorder (FGA [*n* = 0]; SGA [*n* = 1]). In the FGA group, five patients had no comorbidities and in the SGA group six patients had no comorbidities.

### Antipsychotics

All the participants were on antipsychotic drugs. The types and distribution of antipsychotics are shown in Table 2.

**TABLE 2:** First-generation antipsychotics and second-generation antipsychotics prescribed for male inpatients with severe mental illness at Weskoppies Psychiatric Hospital, South Africa.

FGA	*n*	SGA	*n*
Zuclopenthixol decanoate	9	Amisulpride	1
Haloperidol	1	Risperidone	4
Flupentixol decanoate	3	Olanzapine	2
Zuclopenthixol decanoate and haloperidol	2	Clozapine	6
-	-	Quetiapine	1
-	-	Olanzapine and clozapine	1

**Total**	**15**	**Total**	**15**

### Body measures

Participants in the FGA and SGA groups had similar body measures for HC, WC, WHR, WHtR and BMI’s between the two groups (Table 3).

**TABLE 3:** Body measures of male inpatients with severe mental illness, on first-generation antipsychotics and second-generation antipsychotics, at Weskoppies Psychiatric Hospital, South Africa.

Variables	FGA			SGA			Overall			*p*
	Mean	95% CI	Median (IQR)	Mean	95% CI	Median (IQR)	Mean	95% CI	Median (IQR)	
Weight	83.3	73.67, 93.93	81	83.3	75.27, 91.40	63	83.6	77.50, 89.63	81.5	0.94
Height	1.72	1.67, 1.76	1.7	1.7	1.67, 1.74	1.68	1.71	1.68, 1.74	1.7	0.62
Hip circumference	100.07	96.18, 103.96	99	100.8	96.39, 105.27	101	100.5	97.68, 103.22	99	0.78
Waist circumference	100.03	92.99, 107.07	99	101.1	95.22, 106.98	103	100.6	96.26, 104.87	100.8	0.80
Waist-to-hip ratio	1	0.94, 1.06	0.98	1	0.97, 1.03	1	1	0.97, 1.03	0.99	0.88
Waist-to-height ratio	0.58	0.54, 0.62	0.6	0.59	0.56, 0.62	0.61	0.59	0.56, 0.61	0.6	0.59
Body mass index	28.33	25.25, 31.42	26.62	28.5	26.43, 30.73	28.3	28.5	26.69, 30.22	28	0.89

CI, confidence interval; IQR, interquartile range.

## Discussion

Body measures associated with CVD risk in a population of long-term male inpatients who had been admitted for several years and were relatively stable on treatment, on either FGAs or SGAs were done. Participants in our study had similar body measures and similar high risk for CVD. These long-term patients had relatively similar lifestyles, including a similar level of activity, diet and smoking habits. We would thus expect that these patients would have similar body measures, and that any differences could be attributed to factors other than lifestyle, such as prescription medication.

In our study, participants had very similar body measures, irrespective of taking FGAs or SGAs. The two groups were also similar in terms of the presence of comorbidities such as hypertension, diabetes, and hypercholesterolaemia. In the literature, SGAs are generally expected to have more metabolic side effects that increase the risk of CVDs than FGAs.^25,26,27^ In our study, the lack of difference in body measures and CVD risk may be explained by the variety of SGAs prescribed to participants. Second-generation antipsychotics are a heterogenous group of drugs with different metabolic effects. That being so, some SGAs may not be worse culprits than FGAs regarding cardiovascular risk. This has been suggested in a systemic review and meta-analysis done in 2019.^22^ Other studies have suggested that overall cardiovascular risk in patients with SMI may be because of a genetic predisposition.^28^ Clinicians usually assess CVD risk before initiating antipsychotic medication per the recommendations,^9^ resulting in the tendency for patients with high CVD risk being initiated on FGAs, while patients with low CVD risk tend to be initiated on SGAs. This practice may even out CVD risk between the two groups, possibly explaining the similarities in body measures and CVD risk. In our study, participants’ body measures indicated significant CVD risk. The WHO states that men who have a WC greater than 94 are at risk for CVD.^24^ In our study, men had an average WC of 100.6 cm, indicating high cardiovascular risk. Men in our study had greater WCs than men in another South African study that found a WC of greater than 86 cm to be predictive of at least two components of metabolic syndrome.^29^ Men in our study had HCs of almost 100 cm. While larger HC is protective against CVD in women, it seems to have no effect on CVD risk in men.^30^ However, when considered together with WC (but not as a ratio measure), WC and HC have been found to improve risk prediction models for CVD.^31^ A possible explanation for this is that WC values indicate subcutaneous and visceral adipose tissue while HC is a more specific measure of subcutaneous adipose tissue, albeit in a different location in the body.^31^ When considered together, these body measures give a more accurate predictive value than either WC or HC on its own. That being so, the exact interplay between HC and WC needs further clarification.^31^

Other body measure values in our study also suggested higher CVD risk. The participants in our study had a mean BMI of > 25 kg/m^2^ and < 30 kg/m^2^ indicating overweight and increased risk of CVD mortality.^32^ The mean WHtR was 0.59, which is greater than the cut-off value of 0.5, the threshold for increased CVD risk.^15^

### Strengths and limitations

Strengths: We assessed two groups of patients with SMI, either on FGAs or on SGAs, who had similar living circumstances and life styles. We were thus able to account for some confounding variables when comparing potential changes in body measures because of side effects of drugs. We only included men, thus avoided having to control for female metabolic differences. This is also a limitation as it reduces the generilisability of our study to male mental healthcare users on antipsychotic medication and excludes females.

Limitations: Our study was a cross-sectional study, which means that both the exposure and the outcome were assessed simultaneously. We did not account for the length of time patients had been admitted and could not assess the temporal relationship between exposure and outcome. The sample size was small, and there were no adjustments made for the presence of comorbidities. The presence of comorbidities such as hypertesion, diabetes and hypercholesterolaemia was, however, similar in both groups, and it does not seem like their presence made a significant impact on the results of the body measures taken. Finally, we only investigated chronic inpatients with SMI. Outpatients were not included. Because of budget constraints, we did not compare all the metabolic parameters of patients on either FGAs or SGAs.

### Implications and recommendations

Because of the small sample size, generalisation of the results is inappropriate. Nonetheless, it contributes to research drawing on body measures to gauge cardiovascular risk in patients with SMI, who are on antipsychotic drug treatment. Further studies are needed to assess the usefulness of body measures associated with cardiovascular risk in chronic patients with SMI who are treated with FGAs or SGAs.

## Conclusion

In our study, all the participants had similar body measures irrespective of using FGAs or SGAs. Overall the body measures reflected that this sample of chronic, male inpatients with SMI is at high risk for CVD.
